# An Investigation of the Basic Physics of Irrigation in Urology and the Role of Automated Pump Irrigation in Cystoscopy

**DOI:** 10.1100/2012/476759

**Published:** 2012-05-15

**Authors:** Dwayne Chang, Rustom P. Manecksha, Konstantinos Syrrakos, Nathan Lawrentschuk

**Affiliations:** ^1^Urology Unit, Department of Surgery, University of Melbourne, Austin Health, Melbourne, VIC, Australia; ^2^Ludwig Institute for Cancer Research, Austin Health, Melbourne, VIC, Australia; ^3^Department of Surgery, Austin Hospital, 145 Studley Road, P.O. Box 5555, Heidelberg, VIC 3084, Australia

## Abstract

*Objective*. To investigate the effects of height, external pressure, and bladder fullness on the flow rate in continuous, non-continuous cystoscopy and the automated irrigation fluid pumping system (AIFPS). *Materials*. Each experiment had two 2-litre 0.9% saline bags connected to a continuous, non-continuous cystoscope or AIFPS via irrigation tubing. Other equipment included height-adjustable drip poles, uroflowmetry devices, and model bladders. *Methods*. In Experiment 1, saline bags were elevated to measure the increment in flow rate. In Experiment 2, saline bags were placed under external pressures to evaluate the effect on flow rate. In Experiment 3, flow rate changes in response to variable bladder fullness were measured. *Results*. Elevating saline bags caused an increase in flow rates, however the increment slowed down beyond a height of 80 cm. Increase in external pressure on saline bags elevated flow rates, but inconsistently. A fuller bladder led to a decrease in flow rates. In all experiments, the AIFPS posted consistent flow rates. *Conclusions*. Traditional irrigation systems were susceptible to changes in height of irrigation solution, external pressure application, and bladder fullness thus creating inconsistent flow rates. The AIFPS produced consistent flow rates and was not affected by any of the factors investigated in the study.

## 1. Introduction

Optimal visualisation is important in urological endoscopic procedures. Effective fluid irrigation systems are essential for such visualisation by maintaining a clear operative field, improving scope manoeuvrability and enabling organ dilation, which creates further space, which all contribute to improve operative precision and efficiency. Continuous-flow irrigation systems which employ separate simultaneous inflow and outflow channels have been developed in urology which has been found to deliver superior irrigation compared to conventional noncontinuous flow systems [1]. Continuous-flow systems have also led to reduced procedure times due to the enhanced visibility and an improved working space [2]. Traditionally, cystoscopic irrigation is gravity driven and has the disadvantage of having intermittent and occasionally poor flow. Even with continuous-flow instrumentation, manoeuvres such as pressure compression to irrigation fluid bags may be required but this is inconsistent and hampers monitoring of volumes and pressures generated within the bladder.

One advance that may assist in regulating consistent irrigant fluid flow is the automated irrigation fluid pumping system (AIFPS). Such systems have been shown to produce significantly better visibility than gravity-driven irrigation in certain arthroscopic procedures [3]. They allow for control of flow and in some instances the pressure generated within the target organ.

Governing the flow of fluids through any closed system is determined by physics (Box 1) whereby the flow will increase if there is ((3)) an increase in the pressure difference and radius of the tube or ((5)) a decrease in the length of the tube and viscosity of the fluid [4]. Although an increase in the height of the irrigation fluid logically results in a higher pressure difference and thus an increase in flow, the question to be considered is whether there is a reduction in the rate of increase in flow rate after a certain height, thereby decreasing the effect of further increases in the height of irrigation fluid. Furthermore, what is poorly understood and studied is the effect of pressure generated in the bladder when irrigation is used in a closed system or in a continuous flow system, with or without automated pumping systems.

With this background, our aim in this study is to compare the flow properties of traditional gravity-based irrigation versus automated systems (AIFPS), focusing on a cystoscopic setting. The variables to be investigated are the height and application of external pressure on the irrigant bag and the effect of bladder fullness on flow rate.

## 2. Materials and Methods

### 2.1. General Equipment

The study utilised standard 21F non-continuous and continuous flow cystoscopes (Olympus Australia Pty Ltd, Mount Waverly, IC, Australia) for flow experiments. The cystoscopes were then connected via standard irrigation tubing to fluid reservoirs being two 2-litre bags of 0.9% saline solution attached to height adjustable drip poles at two different, fixed levels. Flow rates of fluid emanating from the cystoscope were measured with an Uroflow (*Urocap III *, Laborie Medical Technologies, Toronto, Canada) device.

### 2.2. Irrigant Fluid Reservoirs

In Experiments 1 and 2 (see below), half-full irrigant bags were reused to investigate the effect of reduced irrigant bag content on flow rates. In Experiment 3, new bags of fluid were utilised to overcome the effect of loss of fluid from a bag which may affect the flow properties of the experiments.

### 2.3. Automated Irrigation Fluid Pumping System (AIFPS)

The AIFPS device (ACI pump Dyonics 25 fluid management system, Smith and Nephew, London UK) was configured and used for both gravity-driven irrigation and automated continuous irrigation (ACI) experiments in order to achieve consistency within the study (Figure 6). Pressure generated within the target organ (model) was also measured by the AIFPS device. Prior to experiments, the flow rate was tested to ensure that with gravity, flow rate at each height of the irrigant bag was not altered by passing fluid through the pump and they were not as recorded by our equipment. The AIFPS device was placed at 100 cm above the operating room floor in all cases.

### 2.4. Model Bladders

The most consistent and reproducible model bladder we obtained was a “classic” hot water bottle (500 mL, ribbed). For all experiments these were placed at a height of 100 cm above the operating room floor to simulate a patient's position on an operating table. The bottles were utilised because they have a reasonable degree of compliance felt to be reflective of human bladders and they had an ideal fit with the cystoscope.

### 2.5. Experiments

**(1) Flow Rates for Different Heights of Irrigant Bags**

The flow rates were recorded for a series of different irrigant bag heights. They started from 0 cm (the level of the patient, i.e., 100 cm from the floor) and elevated in increments of 20 cm up to a height of 140 cm above the patient. These experiments were then repeated using irrigant bags that were half full.

**(2) Flow Rates for Different External Pressures on Irrigant Fluid Reservoirs**

From a standardised height of 100 cm above the patient level, the flow rates were recorded for a series of external pressure (manual, 100 mmHg, 220 mmHg, and 300 mmHg) applied to the irrigant bags either manually or via pressure cuff. This was done to simulate pressure applied to irrigation bags during surgery on certain occasions. For each set of pressure, two measurements of flow rates were taken here: one when a full reservoir bag was emptied to half-full and another when a half-full bag was fully emptied.

**(3) Flow Rates into Model Bladders Containing Different Amounts of Fluid**

From a standardised height of 100 cm above the patient level, flow rates were recorded for a series of model bladders with variable amount of irrigant fluid in them (empty, quarter-full, half-full, and near-full model bladders).

### 2.6. Statistical Analysis

Data was entered into a spreadsheet and analyzed using Graphpad Prism 4 (GraphPad Software Inc., La Jolla, CA, USA). The one-way ANOVA analysis was used to compare across groups.

## 3. Results

**Experiment 1 (Effect of Variable Heights of Irrigant Bags on Flow Rate)**

**Figure 1:**
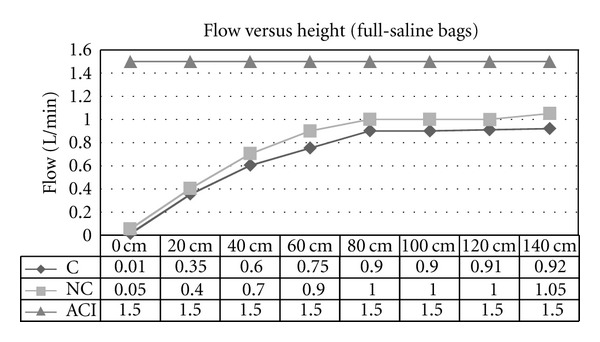
Flow versus height using gravity-controlled irrigation for continuous (C) flow and non-continuous (NC) cystoscopes and ACI device (ACI; set at 1.5 L/min). Setting was when irrigant bags were full to half full.

**Figure 2:**
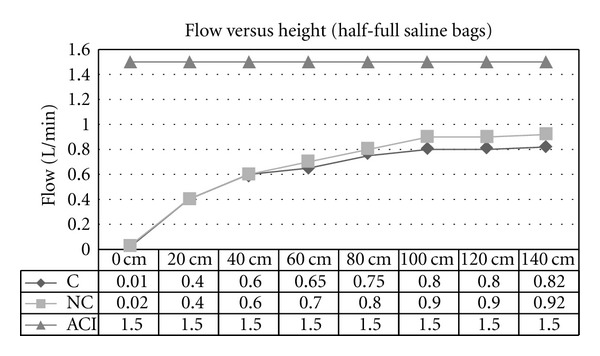
Flow versus height using gravity-controlled irrigation for continuous (C) and non-continuous (NC) cystoscopes and ACI device (ACI; set at 1.5 L/min). Setting was when irrigant bags were less than half-full.

The flow rates increased in proportion with the height of the saline bags for both noncontinuous and continuous cystoscopes (Figure 1). The flow rate reached a maximum of approximately 1.0 L/min at a height of 80 cm. Beyond this height the rate increased nominally at 140 cm height. The same observable effect could be seen in the half full saline bags. Both continuous and non-continuous cystoscopes recorded elevated flow rates with height increase. The maximum flow rate was 0.8 L/min (Figure 2). In both experiments with half and full saline bags, the ACI system maintained a constant flow rate of 1.5 L/min regardless of the height of the bag. In addition, the analysis of variance (ANOVA) statistical model showed strong evidence (*P*-values of 0.0005) to suggest that a true variation exists between the results achieved for the continuous and non-continuous cystoscopes and the ACI system for the flow versus height experiments (Tables 1 and 2).

**Experiment 2 (Effect of Variable External Pressures on Irrigant Bags on Flow Rate)**

**Figure 3:**
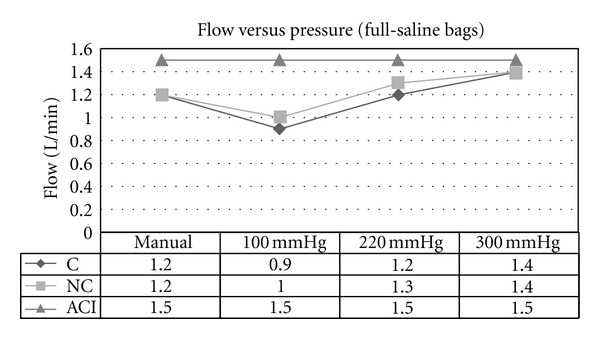
Flow versus pressure using gravity-controlled irrigation for continuous (C) and non-continuous (NC) cystoscopes and ACI device (ACI; set at 1.5 L/min). Setting was when irrigant bags were full to half full.

**Figure 4:**
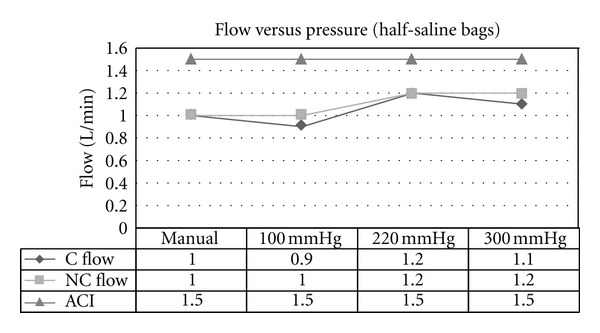
Flow versus pressure using gravity-controlled irrigation for continuous (C) and non-continuous (NC) cystoscopes and ACI device (ACI; set at 1.5 L/min). Setting was when irrigant bags were less than half-full.

A maximally increased flow rate of 1.4 L/min at a cuff pressure of 300 mm Hg, on full irrigant bags was achieved (Figure 3). Flow rates increased with increasing pressure on the irrigant bags, regardless of whether the bags were full (Figure 3) or half-full (Figure 4). External manual pressure was variable, although it appears that the flow rate generated from it is equal or greater than the flow rate produced by an external pressure of 100 mm Hg. In both experiments with half and full saline bags, the ACI system maintained a constant flow rate of 1.5 L/min. Furthermore, the ANOVA statistical model showed that there is strong evidence (*P* values of 0.013 and 0.0008) to suggest that a true variation exists between the results achieved for the continuous and non-continuous cystoscopes and the ACI system for the flow versus pressure experiments (Tables 3 and 4).

**Experiment 3 (Effect of Variable Fullness of Model Bladder on Flow Rate)**

**Figure 5:**
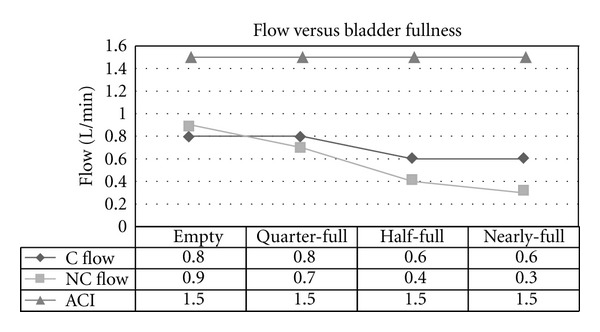
Flow versus bladder fullness using gravity-controlled irrigation for continuous (C) and non-continuous (NC) cystoscopes and ACI device (ACI; set at a pressure of 60 mmHg and flow rate of 1.5 L/min).

**Figure 6:**
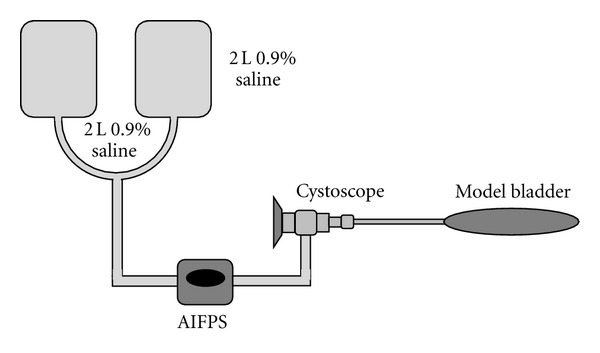
Illustration of the experimental model (not drawn to scale).

The flow rate of the continuous and non-continuous cystoscopes decreased as the amount of fluid in the model bladders increased (Figure 5). This may reflect the increased pressure building up in the model bladders that caused a reduced pressure difference between the irrigant bag and the model bladder, thus causing a decreased flow rate through the cystoscope. The ACI model was maintained at a pressure of 60 mm Hg and flow rate of 1.5 L/min. The ANOVA model also showed strong evidence (*P* value of 0.0005) to suggest that a true variation exists between the results achieved for the continuous and non-continuous cystoscopes and the ACI system (Table 5).

## 4. Discussion

**Figure 7:**
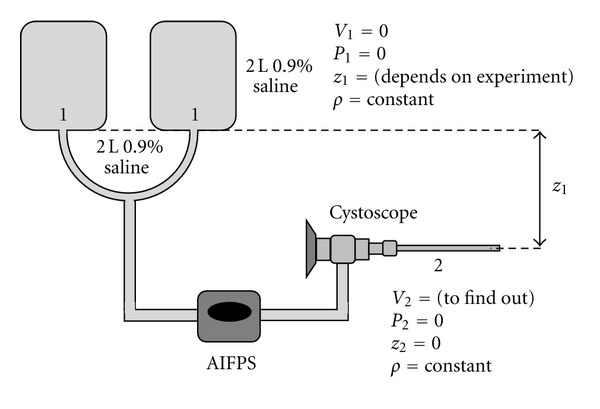
Illustration of the physical properties in the experimental models; *V*: velocity; *P*: pressure; *z*: height; *ρ*: density (constant throughout the experiment).

To assess the effect of height on the flow rate of irrigant, the Bernoulli equation is the ideal equation to be applied [5]. This equation is defined as (refer to the legend in Figure 7 for explanation to mathematical symbols in the equation):

(1) $$z_{1}+\frac{{V_{1}}^{2}}{2g}+\frac{P_{1}}{\rho g}\;=z_{2}+\frac{{V_{2}}^{2}}{2g}+\frac{P_{2}}{\rho g}.$$

In a system where no external pressure is exerted on the irrigant bag and the end of the cystoscope is open (Figure 7), the pressure acting upon the irrigant bag (Point 1) and at the end of the tube (Point 2) is the atmospheric pressure (760 mm Hg), both of which can be considered as 0 (i.e., *P*_1_ and *P*_2_ = 0) to simplify the mathematical process. In addition, the irrigant in the bag is assumed to be still, he

nce it has no velocity (i.e., *V*_1_ = 0). The height at the end of the cystoscope can be considered to be 0 (i.e., *z*_2_ = 0), whereas the height of the irrigant bag (*z*_1_) relative to the end of the cystoscope varies depending on the experiment. These assumptions are essential, in this situation, in order to simplify the equation to the following:

(2) $$z_{1}+\frac{0^{2}}{2g}+\frac{0}{\rho g}=0+\frac{{V_{2}}^{2}}{2g}+\frac{0}{\rho g}.$$

From here, the following formula can be obtained:

(3) $$V_{2}=\;\sqrt[{2}]{z_{1}\left(2g\right)}.$$

**Figure 8:**
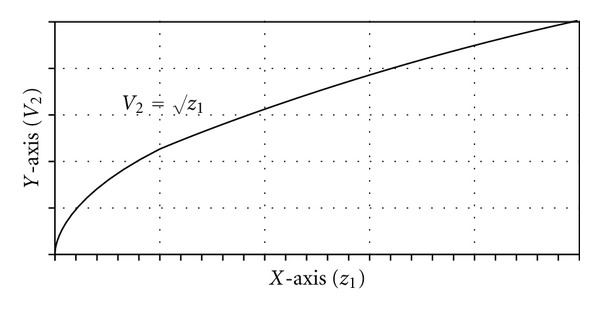
Graph of *V*_2_ versus √*z*_1_.

Formula (3) shows that the velocity of the irrigant at the end of the cystoscope (*V*_2_) is proportional to the square root of the height of the irrigant bag (i.e., √*z*_1_). A graph of *V*_2_ = √*z*_1_ will illustrate their relationship clearly (Figure 8).

Figure 8 demonstrates that although the flow of irrigant at the end of the cystoscope increases with increases in the height of the irrigant bag, the rate of increase in the flow rate of irrigant actually decreases. The graph resembles the results of Experiment 1 (Figures 1 and 2). This suggests that beyond a certain height, the increase in the flow rate of irrigant becomes negligible.

The Bernoulli equation also explains the effect of external pressure on the flow rate of irrigant through a cystoscope. Exerting pressure (either manually or by using a machine) onto the irrigant bag means that the value of *P*_1_ is no longer 0, but rather a positive integer. Thus, by substituting *P*_1_ with a positive integer rather than 0, the Bernoulli equation will appear as

(4) $$z_{1}+\frac{0^{2}}{2g}+\frac{P_{1}}{\rho g}=0+\frac{{V_{2}}^{2}}{2g}+\frac{0}{\rho g}.$$

From here, the following formula can be obtained:

(5) $$V_{2}=\;\sqrt[{2}]{z_{1}\left(2g\right)+\frac{2P_{1}}{\rho }\;}.$$

Once again, Formula (5) shows that the velocity of the irrigant at the end of the cystoscope (*V*_2_) is proportional to the square root of the height of the irrigant bag (*z*_1_). By virtue of the additional positive integer required to calculate the *V*_2_ in Formula (5), it also shows that in situations where there is external pressure acting on the irrigant bag, the flow rate at the end of the cystoscope will be higher than in situations where there is no external pressure.

It is important to acknowledge the effects of other variables that may be present in real-life situations. In actual cystoscopic procedures, the end of the cystoscope does not open to the atmosphere, but rather into the inside of the bladder. This itself may present a problem as the bladder is a closed space and thus with accumulation of irrigant in the bladder during the procedure, the pressure at the end of the cystoscope actually increases while the pressure on the irrigant bag remains the same (atmospheric pressure). Thus *P*_2_ is now a positive integer rather than 0, so the Bernoulli equation now appears as

(6) $$z_{1}+\frac{0{\;}^{2}}{2g}+\frac{0}{\rho g}\;=0+\frac{V_{2}{\;}^{2}}{2g}+\frac{P_{2}}{\rho g}.$$

From here, the following equation can be obtained:

(7) $$V_{2}=\;\sqrt[{2}]{z_{1}\left(2g\right)-\;\frac{{2P}_{2}}{\rho }},$$

By comparing Formulas (3) and (7), it is obvious that if there is an increase of pressure at the end of the cystoscope (*P*_2_), the flow rate of irrigant through the cystoscope (*V*_2_) will decrease. The gradual decrease in flow rates for bladders with increasing fullness in Experiment 3 is a good example of this in practice and is supported by results from a study on arthroscopic irrigation in different degrees of distended joints [6].

The AIFPS consistently maintained rates of 1.5 L/min as the system was able to adjust for any changes in irrigant bag heights. The flow rates of the both continuous and non-continuous were less than that of the AIFPS. The two types of AIFPS generally available are either pressure-only controlled systems or controlled pressure and flow. With the first type, pressure is controlled but flow rate is variable. The second system allows for the control of both pressure and flow but employs a more complex setup. One study found that the pressure- and flow-controlled arthroscopes were superior in terms of visualisation, procedure times, and better safety compared to pressure-only systems [7, 8]. Arthroscopic pump irrigation systems in another study found a low complication rate of 1.5% over 15 months [9].

The results of the gravity flow systems with the external pressure systems resulted in variable flow rates. In some cases it could be concluded that increased pressure did increase flow rates; however, on some occasions the flow rate did not rise in accordance with the level of pressure. This demonstrates the inconsistent effects of external pressure application. The AIFPS on the other hand maintained a constant predictable flow rate of 1.5 L/min despite changes in fluid volume, height, and external pressure thus showing that the AIFPS may have an advantage in terms of visibility over the gravity-based systems. This was supported by an experimental study that showed that for certain arthroscopic procedures, visibility in automated pumping systems as significantly less affected by intra-operative bleeding as compared to gravity-based infusion systems [3].

The main limitation of the study was that the flow measurements were not repeated in view of the numerous readings that needed to be taken in total. Although this may have exposed the final measurements to a higher risk of random errors, the simple experimental setup ensured that there was little chance of them developing so the expected effect of such errors is small.

It can be concluded that irrigation systems that control pressure and flow as independent variable should be considered in cystoscopy as an alternative system. The benefits of constant flow may provide better visualisation and reduced procedure times as seen in arthroscopic procedures [7, 8]. The obvious difference being the lack of compliance in a joint space versus the bladder, but similar issues of visibility still arise in both procedures. One key disadvantage is that controlled irrigation systems require more complex setups which require additional access ports for pressure and flow monitoring and maintenance. In addition, we summarise that the consistent flow may have the benefit of better vision and thus of bleeding but if the consistency lead to larger volumes over many minutes when the bleeding is severe, the surgeon would need to be aware and factor this into the operative management. Also, they must recall that impact on irrigation flow is limited by the scope size and not consistency of flow and patient vital signs and visualisation of irrigant fluid return remain key indicators of blood loss. Another issue is patient safety and associated potential complications when used in cystoscopy (e.g. if the rates were set incorrectly for perhaps a low compliance bladder). Currently there is little information on complication types and the rates from using such systems in urological investigations nor has there been an exploration of additional costs. Finally, alternatives such as using an arthroscopic irrigant-giving set with a simple hand pump operated by the surgeon or assistant may be a useful alternative. However, these are likely to generate only very transient small changes in pressure and flow extrapolating from our studies but these may be all that is required as endoscopic equipment improves through digital technology and smaller design and accompanying assisting equipment.

## 5. Conclusion

From this study, traditional gravity-based systems have demonstrated increased flow rates from increment in the bag volume and height from which the irrigation solution is positioned, but the increase in flow rates was less the higher the position of the irrigation solution was. Manual and inflatable cuff pressure also increased flow rates but were demonstrated to be inconsistent. Urologists should be aware of the limitations of “raising the irrigant bag” and also the inconsistencies in flow and pressure generated by “squeezing the bag.” Automated controlled irrigation systems maintained constant flow rates as an independent variable. Flow rates did not change despite changes in irrigation fluid volume, bag height, external pressure, and bladder fullness. Use in cystoscopic procedures may be of benefit as such systems have documented advantages in other procedures but clinical data is needed to support such hypotheses.

## Figures and Tables

**Box 1:**
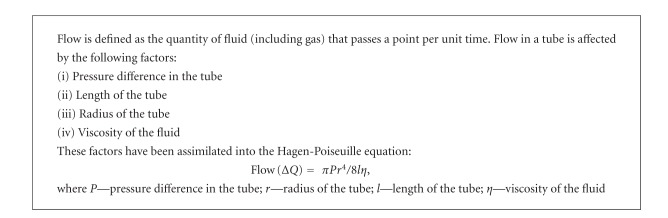
The physics of flow in a tube.

**Table 1:** ANOVA statistical analysis of mean flow rates in Figure 1.

	Mean	Variance	*P* value
C Flow	0.668	0.111	0.0005
NC Flow	0.763	0.131	
ACI	1.5	0.0	

**Table 2:** ANOVA statistical analysis of mean flow rates in Figure 2.

	Mean	Variance	*P* value
C Flow	0.604	0.077	0.0005
NC Flow	0.655	0.098	
ACI	1.5	0.0	

**Table 3:** ANOVA statistical analysis of mean flow rates in Figure 3.

	Mean	Variance	*P* value
C Flow	1.175	0.0425	0.013
NC Flow	1.225	0.0292	
ACI	1.5	0.0	

**Table 4:** ANOVA statistical analysis of mean flow rates in Figure 4.

	Mean	Variance	*P* value
C Flow	1.05	0.017	0.0008
NC Flow	1.1	0.013	
ACI	1.5	0.0	

**Table 5:** ANOVA statistical analysis of mean flow rates in Figure 5.

	Mean	Variance	*P* value
C Flow	0.7	0.013	0.0005
NC Flow	0.575	0.076	
ACI	1.5	0.0	
